# Boron Substituted Na_3_V_2_(P_1_ *_−x_*B*_x_*O_4_)_3_ Cathode Materials with Enhanced Performance for Sodium‐Ion Batteries

**DOI:** 10.1002/advs.201600112

**Published:** 2016-08-02

**Authors:** Pu Hu, Xiaofang Wang, Tianshi Wang, Lanli Chen, Jun Ma, Qingyu Kong, Siqi Shi, Guanglei Cui

**Affiliations:** ^1^ Qingdao Industrial Energy Storage Research InstituteQingdao Institute of Bioenergy and Bioprocess TechnologyChinese Academy of SciencesQingdao266101P. R. China; ^2^ University of Chinese Academy of SciencesBeijing100049P. R. China; ^3^ School of Materials Science and EngineeringShanghai UniversityShanghai200444P. R. China; ^4^ Materials Genome InstituteShanghai UniversityShanghai200444P. R. China; ^5^ Société civile Synchrotron SOLEILL'Orme des MerisiersSaint‐Aubin ‐ BP 4891192Gif‐sur‐Yvette CEDEXFrance

**Keywords:** cathode materials, DFT calculation, doping, Na_3_V_2_(PO_4_)_3_, Na‐ion battery

## Abstract

The development of excellent performance of Na‐ion batteries remains great challenge owing to the poor stability and sluggish kinetics of cathode materials. Herein, B substituted Na_3_V_2_P_3_ *_–x_*B*_x_*O_12_ (0 ≤ *x* ≤ 1) as stable cathode materials for Na‐ion battery is presented. A combined experimental and theoretical investigations on Na_3_V_2_P_3_ *_–x_*B*_x_*O_12_ (0 ≤ *x* ≤ 1) are undertaken to reveal the evolution of crystal and electronic structures and Na storage properties associated with various concentration of B. X‐ray diffraction results indicate that the crystal structure of Na_3_V_2_P_3_ *_–x_*B*_x_*O_12_ (0 ≤ *x* ≤ 1/3) consisted of rhombohedral Na_3_V_2_(PO_4_)_3_ with tiny shrinkage of crystal lattice. X‐ray absorption spectra and the calculated crystal structures all suggest that the detailed local structural distortion of substituted materials originates from the slight reduction of V–O distances. Na_3_V_2_P_3‐1/6_B_1/6_O_12_ significantly enhances the structural stability and electrochemical performance, giving remarkable enhanced capacity of 100 and 70 mAh g^−1^ when the C‐rate increases to 5 C and 10 C. Spin‐polarized density functional theory (DFT) calculation reveals that, as compared with the pristine Na_3_V_2_(PO_4_)_3_, the superior electrochemical performance of the substituted materials can be attributed to the emergence of new boundary states near the band gap, lower Na^+^ diffusion energy barriers, and higher structure stability.

## 1 Introduction

Sodium‐ion batteries (SIBs) are attracting a significant attention as promising alternative to dominant lithium‐ion batteries for the potential application in large‐scale grid storage and electrical vehicles due to the abundant reserves and cost advantages of Na as compared with Li.^1^, ^2^ However, electrochemical performances in terms of rate capability and cycling life associated with SIBs did not come up to those of the lithium counterparts for their practical application. As compared with lithium‐ion, ionic radius of sodium‐ion is much larger, which severely restricts the ion diffusion kinetics of Na^+^ in host material, especially in cathode materials.^3^, ^4^, ^5^, ^6^ Therefore, how to efficiently improve the diffusion kinetics of Na^+^ is a significant issue for development of high performance electrode materials for SIBs.

In the past few years, many compounds including layer‐structured oxide and polyanionic phosphates have been investigated to find a suitable cathode material for reversible and rapid intercalation/deintercalation of Na^+^.^7^, ^8^, ^9^, ^10^, ^11^, ^12^, ^13^, ^14^ Among them, sodium super ion conductor (NASICON) structured Na_3_V_2_(PO_4_)_3_ with 3D open framework and large tunnels for Na^+^ migration has aroused an extensive interest as cathode materials for SIBs owing to its superiority of good stability, moderate potential plateau, high energy density, and so on.^14^, ^15^, ^16^, ^17^, ^18^ However, the poor intrinsic electronic conductivity and structural stability of Na_3_V_2_(PO_4_)_3_ severely retard the cycling stability and rate capability of electrode in its practical application. To overcome such fundamental demerits, several strategies, including coating various carbon materials, reducing the particle size to nanoscale, and doping with the foreign ion, have been widely proposed in literature.^19^, ^20^, ^21^, ^22^, ^23^

Ion‐doping can be considered as an effective way to enhance the electronic conductivity of electrode material for improving the electrochemical performance of lithium and/or sodium ion batteries. Various metallic ions with different valence states and ionic sizes (such as K^+^, Fe^2+^, Mg^2+^, and so on)^23^, ^24^, ^25^ were introduced to partly substitute Na and/or V of Na_3_V_2_(PO_4_)_3_ units, and hence a modified Na_3_V_2_(PO_4_)_3_ with high capacity and excellent rate property can be achieved. So far, research on nonmetal substitution of Na_3_V_2_(PO_4_)_3_ at P‐site and/or O‐site is rare, except some cases on Na_3_V_2_(PO_4_)_3–_ *_x_*F_3_ *_x_*, in which F atoms were utilized to replace the whole PO_4_^3−^.^26^, ^27^, ^28^ Actually, the strategy of doping nonmetal element in polyanion cathode materials of LIBs to improve the electrochemical property has been studied.^29^, ^30^, ^31^, ^32^, ^33^, ^34^ For example, Sin et al. reported that boron polyanion substituted LiFe_0.4_Mn_0.6_(PO_4_)_3_ showed a high initial capacity and low over potential during cycling.^35^ Similar enhancement of electrochemical performance can also be observed in olivine LiMnPO_4_ and LiFePO_4_ cathode, NASICON‐type Li_3_V_2_(PO_4_)_3_ cathode and solid‐state electrolyte LiTi_2_(PO_4_)_3_ with an optimum amount of B.^31^, ^36^ They believed that replacement of B not only introduces the p‐type conductivity, but also significantly increases Li^+^ diffusion kinetics and structure stability.

Inspired by the positive effects of B doped compounds with enhancement of electrochemical performance in LIBs, we herein presented B substituted Na_3_V_2_P_3_ *_–x_*B*_x_*O_12_ (0 ≤ *x* ≤ 1) as cathode materials for SIB. A combined experimental and theoretical study on Na_3_V_2_P_3_ *_–x_*B*_x_*O_12_ (0 ≤ *x* ≤ 1) were undertaken to reveal the evolution of crystal and electronic structure and Na‐ion diffusion properties associated with various amount of B. Electrochemical properties of Na_3_V_2_P_3_ *_–x_*B*_x_*O_12_ were optimized by adjusting B contents. Na_3_V_2_P_3‐1/6_B_1/6_O_12_ shows an obvious enhancement in cycling stability, fast rate capability, and Na‐ion diffusion kinetics. DFT calculations revealed that the enhancement of Na^+^ migration derives from the optimized crystal structure and the decreases of the bad gap are due to the appearance of the new bound states by B substitution.

## 2 Results and Discussion

**Figure 1:**
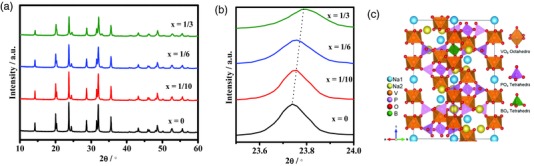
a) XRD patterns of Na_3_V_2_P_3_ *_–x_*B*_x_*O_12_ (x = 0, 1/10, 1/6, 1/3) powders, b) magnified view, and c) calculated crystal structure of Na_3_V_2_P_3_ *_–x_*B*_x_*O_12_ (*x* = 1/6).

Phase purity of the Na_3_V_2_P_3_ *_–x_*B*_x_*O_12_ (0 ≤ *x* ≤1) with various B concentrations was examined by X‐ray diffraction (XRD) patterns. As shown in **Figure** **1**, A series of Na_3_V_2_P_3_ *_–x_*B*_x_*O_12_(0 ≤ *x* ≤ 1/3) with various amount of B can be observed in the rhombohedral crystal structure, which could be well‐indexed into the NASICON Na_3_V_2_(PO_4_)_3_ with R‐3c space group, indicating the substituted B in the range of 0 ≤ *x* ≤ 1/3 did not change the crystal framework of parent material. Moreover, the continuous shift of peak position to higher angle with increased *x* in Na_3_V_2_P_3_ *_–x_*B*_x_*O_12_ (0 ≤ *x* ≤ 1/3) implies a solid solution over this composition range, and a shrink of crystal size affected by the substitution of B. This observation is in agreement with previous report concerning variations of crystal volume of the B doped Li_3_V_2_(PO_4_)_3_.

**Figure 2:**
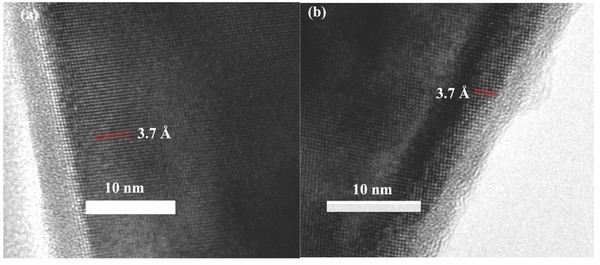
HR‐TEM images of Na_3_V_2_P_3_ *_–x_*B*_x_*O_12_: a) *x* = 0 and b) *x* = 1/6.

Moreover, the half‐height width of the peak changes to broaden with the increasing of B contents. The average crystallite sizes of Na_3_V_2_P*_3–x_*B*_x_*O_12_ with x = 0, 1/10, 1/6, and 1/3 calculated by Scherrer's equation are 205, 110, 80, and 64 nm, respectively, indicate that doping B can prevent the growth of nanoparticles, which is favorable for improving the kinetics of Na^+^ diffusion in bulk materials. Nevertheless, substantial changes in the B substitution in a Na_3_V_2_P_3_ *_–x_*B*_x_*O_12_ (0 ≤ *x* ≤ 1) procure the formation of impurities of Na_2_V(PO_4_)_2_ while *x* > 1/2 (Figure S1, Supporting Information). The particle size and morphologies of Na_3_V_2_P_3_ *_–x_*B*_x_*O_12_(0 ≤ *x* ≤ 1/3) were explored by scanning electron microscopy (SEM) (Figure S2, Supporting Information), showing sphere‐like morphologies with similar particle size range of 0.5–2 μm. The high resolution transmission electron microscope (HR‐TEM) images (**Figure** **2**) of Na_3_V_2_P_3_ *_–x_*B*_x_*O_12_ with *x* = 0 and *x* = 1/6 shows that both particles are coated uniformly by carbon layer with around 3 nm. It is believed that modification with carbon could endow the Na_3_V_2_P_3_ *_–x_*B*_x_*O_12_ higher electronic conductivity. Interplanar spacing of 3.7 Å corresponded to the (113) planes of Na_3_V_2_(PO_4_)_3_.

According to the reports on the ion doping, the incorporation of impurity is more likely to replace those ions which have the similar valence states and the closer ionic radii.^37^ Therefore, the boron prefers to substitute phosphorus or vanadium. To explore the effect of B doped Na_3_V_2_(PO_4_)_3_, a spin‐polarized density functional theory (DFT) calculation was performed to investigate the crystal structure change while phosphorus or vanadium is replaced by boron. Figure 1c shows the lowest total energy configuration of Na_3_V_2_P_3‐1/6_B_1/6_O_12_, in which boron atom occupies the tetrahedral interstitial site. It can be clearly observed that a small amount of boron doping does not alter the framework of the material (Figure S3, Supporting Information). The corresponding lattice constants before and after doping are listed in Table S1 in the Supporting Information. The changes in the lattice parameter of the calculated Na_3_V_2_(PO_4_)_3_ with reference to the experimental value are less than 2%. The optimization results show that the lattice constant of Na_3_V_2_P_3–x_B_x_O_12_ (0 ≤ *x* ≤ 1/3) decreases with the increase of boron concentration and a slight deformation of initial rhombohedral structure, which is owing to the smaller ion radius of B^3+^ (0.27 Å) relative to P^5+^ (0.38 Å). This is in good agreement with the XRD results. In addition, due to the weaker Coulomb interaction, the strength of the B—O bond is weaker than that of the P—O bond which can provide larger Na^+^ diffusion channel. However, the lattice parameter *c* elongates and volume expands significantly when the boron substitutes vanadium site, which are not conform to the experimental results, indicating that boron prefers to substitute for phosphorus.

**Figure 3:**
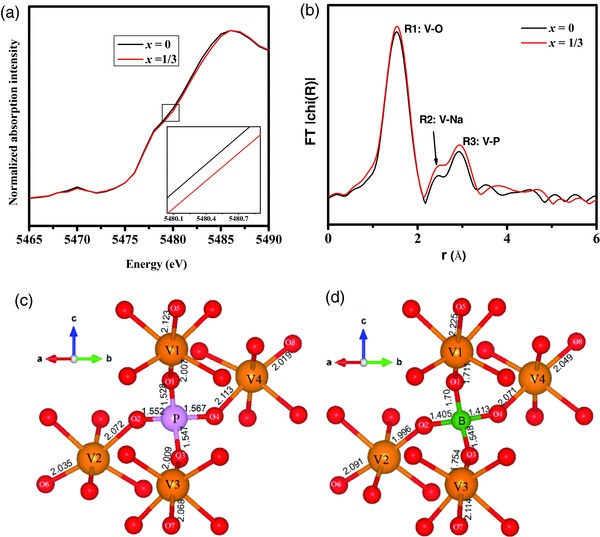
a) XANES and b) Fourier transformed EXAFS spectra of the Na_3_V_2_P_3_ *_–x_*B*_x_*O_12_ (*x* = 0 and *x* = 1/3). Local distortion around the doping site after structural optimization of Na_3_V_2_P_3_ *_–x_*B*_x_*O_12_ c) (*x* = 0) and d) (*x* = 1/6).

To identify the local structural change and enhance the comprehension of the shrink of crystal volume affected by B substitute, X‐ray absorption spectra including X‐ray absorption near‐edge structure (XANES) and extend X‐ray absorption fine structure (EXAFS) were carried out. According to XANES (**Figure** **3**a) at V K‐edge, the edge of B substituted sample shifts to higher energy compared to pristine sample, indicating a higher oxidation state of V in Na_3_V_2_P_3_ *_‐_*_1/3_B_1/3_O_12_, which is consistent with the fact that replacing P^5+^ with aliovalent ion B^3+^ accompanies partly charge compensation of V in the compound. X‐ray photoelectron spectroscope (XPS) of V 2p further confirms the formation of V^4+^ (Figure S4, Supporting Information) and demonstrates that boron successfully a substitute for phosphorus as expected. As Park et al. noted,^38^ V^4+^ usually exhibits a short vanadyl V—O bond length, which trends toward a distorted VO_6_ octahedron. XANES spectra at V K‐edge reveal the local environment surrounding V atom. The variation of peak intensity for the samples may be caused by the distortion of VO_6_ octahedron and other local structural changes after incorporation of B in crystal. Moreover, the V–O distances of the B substituted sample Na_3_V_2_P_3‐1/3_B_1/3_O_12_ and pristine Na_3_V_2_(PO_4_)_3_ determined from the EXAFS data (Figure 3b) was 1.964 Å and 1.971 Å, suggesting that V–O distances were slightly reduced by substitution of B.

The local structure around the doping site was simulated to better understand the variation. As shown in Figure 3c,d, detail local environment for P and B illustrates that the bond length of B‐O2 (O3, O4) in Na_3_V_2_P_3‐1/6_B_1/6_O_12_ is slightly shorter than that of P‐O2 (O3, O4) in the corresponding position of pure Na_3_V_2_(PO_4_)_3_, while the bond length of B‐O1 in Na_3_V_2_P_3‐1/6_B_1/6_O_12_ is slightly longer than that of P‐O1 in pure Na_3_V_2_(PO_4_)_3_, which illustrates the local tetrahedral distortion after B doped Na_3_V_2_(PO_4_)_3_. In addition, as compared with the undoped samples, the V1—O1 and V3—O3 bond length of Na_3_V_2_P_3‐1/6_B_1/6_O_12_ is significantly shortened, while other V—O bond length only slight shrinkage (Table S2, Supporting Information), showing the same tendency with the experimental results. For the pure Na_3_V_2_(PO_4_)_3_ or NaV_2_(PO_4_)_3_, the calculated local magnetic moments of V ion are the same (1.98 or 1.103 μ_B_). However, the calculated local magnetic moment of V1 (1.179 μ_B_) and V3 (1.170 μ_B_) are quite different with other V ion (1.975–1.988 μ_B_) in Na_3_V_2_P_3‐1/6_B_1/6_O_12_, indicating that the V1 and V3 ions are oxidized when boron substitutes phosphorus. Therefore, the strong Coulomb interaction leads to the shrinkage of V1‐O1 and V3‐O3 when the oxidation state of V1 and V3 changes from V^3+^ to V^4+^. The change of local element valence induces the adjacent polyhedron geometry distortion.

**Figure 4:**
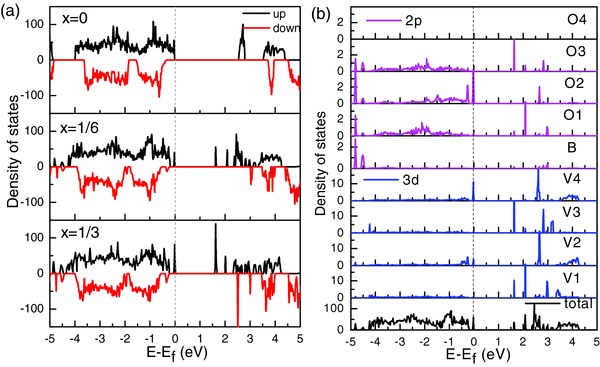
Calculated a) total density of states (DOS) of Na_3_V_2_P_3_ *_–x_*B*_x_*O_12_ (x = 0, 1/6, 1/3) and b) partial spin up density of states of Na_3_V_2_P_3‐1/6_B_1/6_O_12_. E_f_ represents the Fermi energy level.

The effects of substitution on intrinsic electronic conductivity were explored by calculating the density of state of Na_3_V_2_P_3_ *_–x_*B*_x_*O_12_. The spin up density of states (DOS) of Na_3_V_2_(PO_4_)_3_ and the projected DOS of atoms as labeled in Figure 3c are displayed in Figure S5 in the Supporting Information. It clearly shows that Na_3_V_2_(PO_4_)_3_ is a semiconductor with a large band gap of 2.57 eV and the high energy occupied regions in valence band are mainly ascribed to the V‐3d states with little contribution of O‐2p states, while the low energy occupied regions in conduction band are mainly ascribed to the O‐2p states with little contribution of V‐3d states, which are in agreement with the other theoretical calculation result using HSE06 hybrid function.^39^ All the V‐3d states and O‐2p states are nearly the same. Due to the larger band gap, the electronic conductivity of Na_3_V_2_(PO_4_)_3_ is poor, which seriously affects its electrochemical performance. In order to identify the possible electron conduction mechanism after B doping Na_3_V_2_(PO_4_)_3_, the electronic structures of Na_3_V_2_P_3_ *_–x_*B*_x_*O_12_ (*x* = 0, 1/6, 1/3) were calculated. The band gap of Na_3_V_2_P_3_ *_–x_*B*_x_*O_12_ (as listed in Table S1 in the Supporting Information) decreases with the increase of boron doping due to the occurrence of new boundary states, as shown in **Figure** **4**a. However, although the band gap of Na_3_V_2_P_3‐1/6_B_1/6_O_12_ (*E*_g_ = 1.633 eV) is significantly narrower than Na_3_V_2_(PO_4_)_3_, the change is not obvious when continuing to increase the amount of doping. The partial spin up density of states of Na_3_V_2_P_3‐1/6_B_1/6_O_12_ as presented in Figure 4b illustrates that the new energy states in the band gap near the bottom of conduction band are mainly ascribed to the 3d states of V1 and V3 atoms, while those near the top of the valence band are mainly ascribed to the 3d states of V2 and V4 atoms. There are two holes when B^3+^ substituted P^5+^, which may be transferred to the vicinity of the V1 and V3 atoms and induce the new energy states near the bottom of conduction band. Then the oxidation state of V1 and V3 changes from V^3+^ to V^4+^, resulting the VO_6_ octahedral distortion. The change of the local structure also affects the electronic structure of the adjacent atoms, such as V2 and V4, and the appearance of the new energy states may improve the electrical properties of the materials.

**Figure 5:**
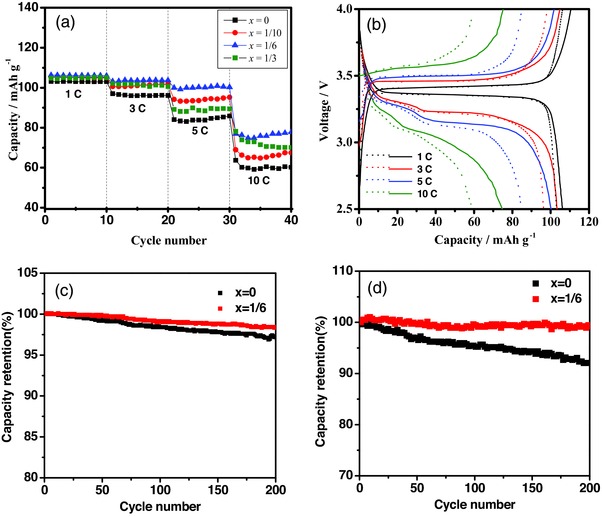
a) Rate capability of the Na_3_V_2_P_3_ *_‐x_*B*_x_*O_12_ (*x* = 0, 1/10, 1/6, 1/3) electrodes at various current density, and charge‐discharge profile of the Na_3_V_2_P_3_ *_‐x_*B*_x_*O_12_ b) (dotted lines for *x* = 0, solid lines for *x* = 1/6), cycling stability of the samples at c) 1 C and d) 5 C.

To investigate the influence of B substitution on the cycling stability and rate capability of the batteries, half‐cells were tested at various charge/discharge rates in the potential range of 2.5–4.0 V. **Figure** **5**a compares the rate capability of the Na_3_V_2_P_3–_ *_x_*B*_x_*O_12_ (*x* = 0, 1/10, 1/6, 1/3) electrodes at charge–discharge rate increasing from 0.5 to 10 C. All the samples display the similar capacity of 105 mAh g^−1^ at lower C‐rate of 1 C regardless of B content. However, samples with various B concentration shows distinct improvement of high C‐rate capability above 3 C. Na_3_V_2_P_3–x_B*_x_*O_12_ with *x* = 1/6 exhibits the highest rate capability, giving remarkable enhanced capacity of 100 and 70 mAh g^−1^ when the C‐rate increases to 5 C and 10 C, respectively. The flat potential plateau displaying nearly theoretical capacity of 2 Na intercalation/deintercalation at around 3.4 V with weak polarization was observed at 1 C (Figure 5b). With the increase of C‐rate, Na_3_V_2_(PO_4_)_3_ delivers the lower capacity and larger potential gap as compared with that of Na_3_V_2_P_3‐1/6_B_1/6_O_12_, indicating more severe polarization at higher C‐rate due to sluggish kinetics of electrochemical reaction.

The cycling performance of the electrode with B substitution was slightly improved than that of pristine Na_3_V_2_(PO_4_)_3_ (Figure 5c). Na_3_V_2_P_3‐1/6_B_1/6_O_12_ maintained 98.4% of the initial discharge capacity after 200 cycles, whereas the Na_3_V_2_(PO_4_)_3_ retained a lower portion of 97.2% of the initial discharge capacity after the same cycles, exhibiting the similar cycling stability of the electrodes. However, the pronounced discrepancy of cycling stability of the samples was observed at an elevated cycling rate of 5 C. As shown in Figure 5d, the capacity retention of Na_3_V_2_P_3‐1/6_B_1/6_O_12_ is 98.7% after 200 cycles, which is much higher than that of pristine Na_3_V_2_(PO_4_)_3_ (91.8%). Capacity retention of the electrodes demonstrates that the electrode with B substitution improves the cycling performance of the battery, especially at higher C‐rate.

**Figure 6:**
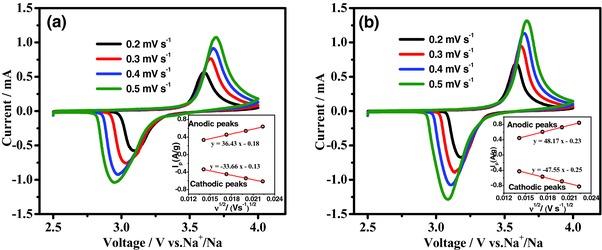
CV curves of the pristine a) Na_3_V_2_(PO_4_)_3_ and b) Na_3_V_2_P_3‐1/6_B_1/6_O_12_ at various sweep rates; b) *I*_p_ versus υ^1/2^ and linear fitting curves of CV.

Further kinetic analysis of the Na_3_V_2_P_3–x_B*_x_*O_12_ (*x* = 0 and *x* = 1/6) was performed by cyclic voltammograms (CV). CV curves at a scan rate of 0.5 mV s^−1^ in potential range of 2.5–4.0 V versus Na^+^/Na (Figure S6, Supporting Information) show that Na_3_V_2_P_3‐1/6_B_1/6_O_12_ exhibits a smaller potential polarization between cathodic and anodic peaks than pristine material, indicating weaker electrode polarization and faster ionic migration. With increasing of scan rates from 0.2 to 0.5 mV s^−1^ (**Figure** **6**), slight shifts of potential of oxidation and reduction peaks at fast scan rate further suggest the existence of fast kinetics and weak polarization of the electrochemical reaction for electrode. A linear relationship between peak current (i_p_/m) and square root of potential scan rate indicates that the electrode reaction would be favored to be a diffusion‐determining step. Diffusion coefficient of Na ions (D_Na_) can be calculated from the slope of the linear relationship using Randles–Sevcik equation

$$i_{p}\mathrm{/m}=0.4463{\left(F^{3}\mathrm{/RT}\right)}^{1/2}n^{3/2}{\mathrm{AD}}^{1/2}C_{0}v^{1/2}.$$

The absolute values of slope for anodic and cathodic peaks of Na_3_V_2_P_3‐1/6_B_1/6_O_12_ are 48.17 and 47.55, higher than that of pristine Na_3_V_2_(PO_4_)_3_ (36.43 and 33.66) from the linear fitting results. Therefore, the D_Na_ is ≈1.7 and 1.9 times higher in Na_3_V_2_P_3‐1/6_B_1/6_O_12_ than those in pristine Na_3_V_2_(PO_4_)_3_ for anodic and cathodic process, respectively. Fast kinetics properties can be attributed to the enhanced ionic and electronic conductivities of the materials after B substitution.

**Figure 7:**
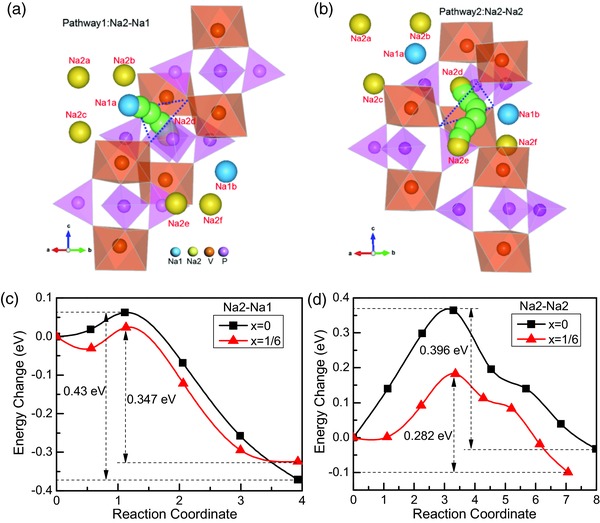
The calculated diffusion trajectory of Na ion along a) pathway 1 and b) pathway 2 in Na_3_V_2_(PO_4_)_3_, and the corresponding diffusion energy barriersalong conduction path c) Na2‐Na1 and d) Na2‐Na2 in Na_3_V_2_P_3_ *_–x_*B*_x_*O_12_ (*x* = 0 and *x* = 1/6). The light green balls, connecting the two Na atoms, emphasize the tracks of diffusion. The blue dashed lines represent the bottleneck triangle.

Na_3_V_2_(PO_4_)_3_ has two Na ion positions, namely Na1 and Na2, along the conduction channels and there are two typical ion transport pathways in the NASICON structure, namely Na2‐Na1‐Na2 and Na2‐Na2.^40^ In order to figure out the effect of doping on ionic conduction, the Na ion hopping barriers along the path of Na2‐Na1 and Na2‐Na2 in Na_3_V_2_P_3_ *_‐x_*B*_x_*O_12_ (*x* = 0 and *x* = 1/6) were calculated using the climbing image nudged elastic band (CINEB) method. The distances of neighboring Na1‐Na2 (3.315 Å) and Na2‐Na2 (4.358 Å) near the doping site in Na_3_V_2_P_3‐1/6_B_1/6_O_12_ are shorter than those in Na_3_V_2_(PO_4_)_3_ (3.416 Å, 4.472 Å) respectively; however, the distances of Na1‐Na2 and Na2‐Na2 far from the doping site in Na_3_V_2_P_3‐1/6_B_1/6_O_12_ are nearly the same as those in Na_3_V_2_(PO_4_)_3_. The diffusion trajectory in Na_3_V_2_(PO_4_) and the corresponding migration energy barriers for Na ion near the doping site along the path of Na2‐Na1 and Na2‐Na2 in Na_3_V_2_P_3‐_ *_x_*B*_x_*O_12_ (*x* = 0 and *x* = 1/6) are plotted in **Figure** **7**. In pathway 1 and pathway 2, the Na ions diffuse through a bottleneck triangle and the migration trajectory are parallel to the two nearest VO_6_ octahedral due to the strong electrostatic repulsion between V and Na ions (Figure 7a,b). Although the activation energy along Na2–Na2 (0.396 eV) is lower than that of Na2–Na1 (0.43 eV), the distance of Na2–Na2 is relative longer. The diffusion routes of Na ion in Na_3_V_2_P_3‐1/6_B_1/6_O_12_ are similar to that in Na_3_V_2_(PO_4_)_3_. As discussed above, the shrinkage of tetrahedral of the doping site and the distortion of adjacent octahedral after B doping Na_3_V_2_(PO_4_) may lead to a larger diffusion path. The energy barriers of Na ion in Na_3_V_2_P_3‐1/6_B_1/6_O_12_ along Na2‐Na1 (0.347 eV) and Na2‐Na2 (0.282 eV) are lower than that in Na_3_V_2_(PO_4_)_3_ (Figure 7c,d), indicating that Na ion is more likely to migrate after B substituted P as a result of the shorter distance of Na1–Na2 and Na2–Na2 and the larger diffusion channel induced by the local structure distortion.

## 3 Conclusion

A series of B substituted Na_3_V_2_P_3_ *_–x_*B*_x_*O_12_ (0 ≤ *x* ≤ 1) have been successfully prepared by a sol–gel process. The XRD and XAS characterizations demonstrated that B doping did not change the crystal framework of parent material in the range of 0 ≤ *x* ≤ 1/3, but aroused the local structure distortion with tiny shrink of crystal lattice. Confirmed from the results of DFT calculation, doping of B in material could change local element valence, resulting in the adjacent polyhedron geometry distortion, which narrow the band gap and facilitate the diffusion of Na^+^. B substituted Na_3_V_2_P_3_ *_–x_*B*_x_*O_12_ significantly enhacnes the structure stability and electrochemical performance. Na_3_V_2_P_3‐1/6_B_1/6_O_12_ exhibits the best cycling stability and rate capability. The capacity retention of Na_3_V_2_P_3‐1/6_B_1/6_O_12_ (98.7%) is much higher than that of pristine materials (91.8%) after 200 cycles at 5 C.

## 4 Experimental Section

*Computation Methods*: First‐principles spin‐polarized density functional theory (DFT) calculations^41^ have been performed to investigate the Boron doping to the NASICON structured Na_3_V_2_(PO_4_)_3_ cathode materials using the Vienna ab initio simulation package^42^ with the projector augmented waves (PAW) pseudopotentials^43^ and the Perdew–Burke–Ernzerhof (PBE) exchange‐correlation functional.^44^ The plane‐wave basis set was determined with a cutoff energy of 520 eV and the k‐point sampling of Brillouin‐zone integrals used a 2 × 2 × 1 Gamma grid. The simplified rotationally invariant approach introduced by Dudarev^45^ to the Hubbard model corrections was used with a U‐J parameter of 4.2 eV for vanadium atom, whereas the correction was not used for the other species. The U value of vanadium is a little higher than the experimental formation enthalpy of vanadium oxides as there are high inductive effects in vanadium phosphates.^46^ The calculations were performed in a rhombohedral chemical cell consisting 6‐f.u. Na_3_V_2_(PO_4_)_3_ and a sodium‐ordered form was chosen in our models which had been demonstrated in other literatures.^47^, ^48^ Different boron substitution positions of Na_3_V_2_P_3_ *_–x_*B*_x_*O_12_ (*x* = 1/6 and *x* = 2/6) and Na_3_V_2‐1/6_B_1/6_(PO_4_)_3_ were calculated, and the lowest energy structure was selected. The Na^+^‐ion hopping barriers were obtained using the climbing image nudged elastic band (CINEB) method, which can determine the pathway for structure changes efficiently.

*Sample Preparation*: The NASICON structured Na_3_V_2_P_3_ *_–x_*B*_x_*O_12_ (0 ≤ *x* ≤ 1) was synthesized by a sol–gel process and followed by solid‐state reaction. Typically, 2 mmol NH_4_VO_3_ and 4 mmol citric acid were added to 70 mL deionized water maintaining at 80 °C with continuous stirring to obtain a clear solution, and then stoichiometric amount of Na_2_CO_3_, NH_4_H_2_PO_4_, and H_3_BO_3_ were added. After evaporation of water at 80 °C, the solution transforms from sol to gel. The gel was dried in an oven at 150 °C for 4 h, and heat‐treated at 400 °C for 5 h under nitrogen atmosphere to remove CO_2_, H_2_O, and NH_3_. Afterward, the powder was grounded and annealed at 800 °C under Ar flow for 12 h to produce the final compound.

*Structure Characterization*: X‐ray diffraction (XRD) patterns of the as prepared samples were collected on a Bruker D8 diffractometer using Cu Kα radiation. The morphology of the materials was observed by Hitachi S‐4800 field emission scanning electron microscope (FE‐SEM). High resolution transmission electron microscope (HR‐TEM) images were taken on a JEOLJEM‐2010F microscope (JEOL, Japan) at an acceleration voltage of 200 kV. X‐ray absorption spectroscopy (XAS) was measured at the V k‐edge, at 12‐BM at the advanced photon source (APS), Argonne national laboratory.

*Electrochemical Characterization*: The work electrode was prepared by coating a mixture of 80 wt% Na_3_V_2_P_3_ *_–x_*B*_x_*O_12_ (0 ≤ *x* ≤ 1), 10 wt% PVDF and 10 wt% Super P onto Al current collector. The electrode was then dried at 120 °C in vacuum for 12 h. The test cells were assembled into coin cell in an argon‐filled glovebox. Sodium metal foil was used as the counter electrode and a glass fiber filter (Whatman) was used as the separator. A 1 m solution of NaClO_4_ in EC/DMC (1:1 by volume) was prepared and used as the electrolyte. The charge/discharge, C‐rate capacity and cycling ability of cells were recorded on a LAND battery test system. Cyclic voltammograms (CVs) were performed using a CHI 440A instrument (CHI Instrument Inc.) at a scanning rate from 0.2 to 0.6 mV s^−1^.

## Supporting information

As a service to our authors and readers, this journal provides supporting information supplied by the authors. Such materials are peer reviewed and may be re‐organized for online delivery, but are not copy‐edited or typeset. Technical support issues arising from supporting information (other than missing files) should be addressed to the authors.

Supplementary
