# Supplementary material for: Boron Substituted Na3V2(P1 −xBxO4)3 Cathode Materials with Enhanced Performance for Sodium‐Ion Batteries
Source: Adv Sci (Weinh). 2016 Aug 2;3(12):1600112. doi: 10.1002/advs.201600112 (PMC5157167; doi:10.1002/advs.201600112)
Supplement: Supplementary file 1 — Supplementary [file ADVS-3-0-s001.pdf]

Copyright WILEY-VCH Verlag GmbH & Co. KGaA, 69469 Weinheim, Germany, 2016.

ADVANCED SCIENCE
Open Access

Supporting Information

for *Adv. Sci.,* DOI: 10.1002/advs.201600112

Boron Substituted $Na_3V_2(P_{1-x}B_xO_4)_3$ Cathode Materials with Enhanced Performance for Sodium-Ion Batteries

Pu Hu, Xiaofang Wang, Tianshi Wang, Lanli Chen, Jun Ma, Qingyu Kong, Siqi Shi,* and Guanglei Cui**

Supporting information

Boron Substituted $Na_3V_2(P_{1-x}B_xO_4)_3$ Cathode Materials with Enhanced Performance for Sodium-ion Batteries

*Pu Hu,Xiaofang Wang,Tianshi Wang, Lanli Chen, Jun Ma, Qingyu Kong,*Siqi Shi,* and Guanglei Cui**

P. Hu, T.-S. Wang, Dr. J. Ma, Prof. G.L. Cui

Qingdao Industrial Energy Storage Research Institute, Qingdao Institute of Bioenergy and Bioprocess Technology, Chinese Academy of Sciences, Qingdao 266101, P. R. China

E-mail: cuigl@qibebt.ac.cn

X. F. Wang, L.L. Chen, Prof. S.Q. Shi

School of Materials Science and Engineering, Shanghai University, Shanghai 200444, P. R. China

Materials Genome Institute, Shanghai University, Shanghai 200444,P. R. China

E-mail: sqshi@shu.edu.cn

Dr. Q.Y. Kong

Société civile Synchrotron SOLEIL, L'Orme des Merisiers, Saint-Aubin - BP 48, 91192 GIF-sur-YVETTE CEDEX, France

E-mail: kong@synchrotron-soleil.fr

P. Hu

University of Chinese Academy of Sciences, Beijing 100049, P. R.China

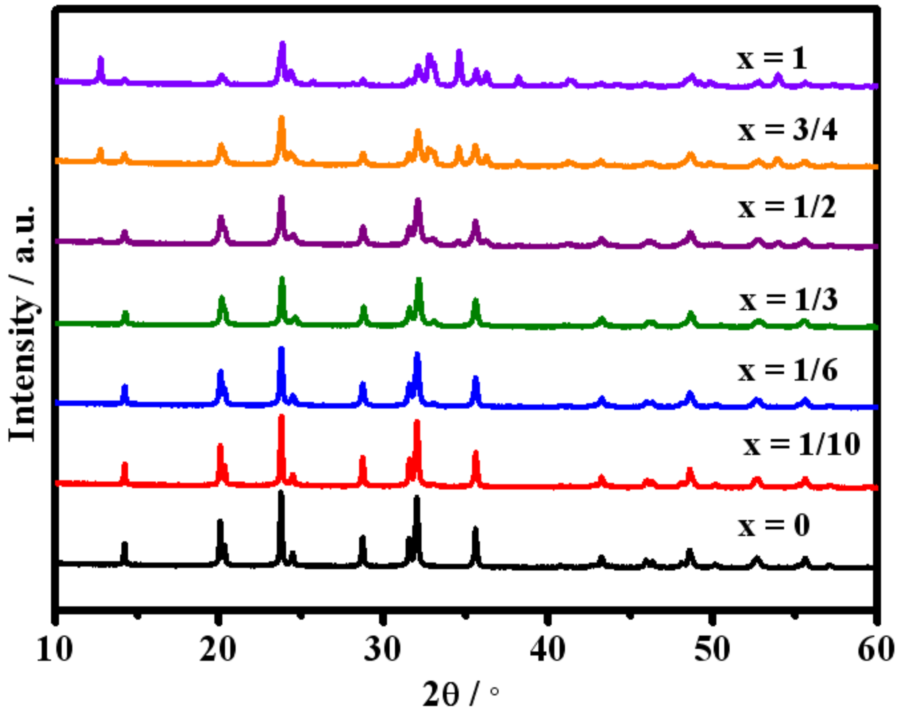

Figure S1. XRD patterns of $Na_3V_2P_{3-x}B_xO_{12}$ ($0 \leq x \leq 1/3$) powders

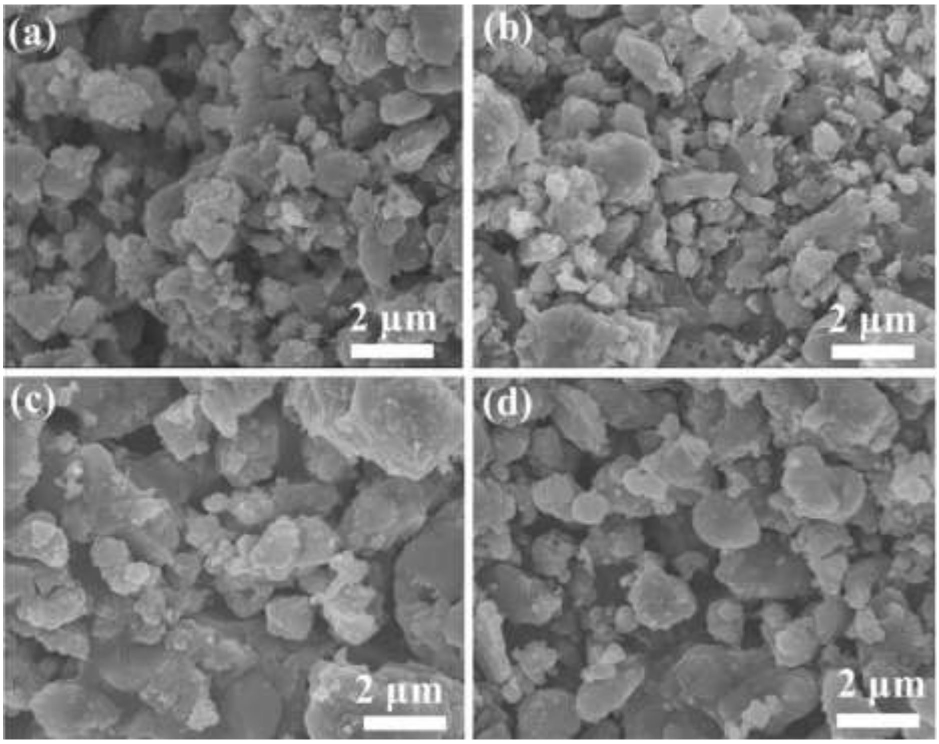

Figure S2.SEM images of $Na_3V_2P_{3-x}B_xO_{12}$: x = 0 (a), x = 1/10 (b), x = 1/6 (c), x = 1/3 (d).

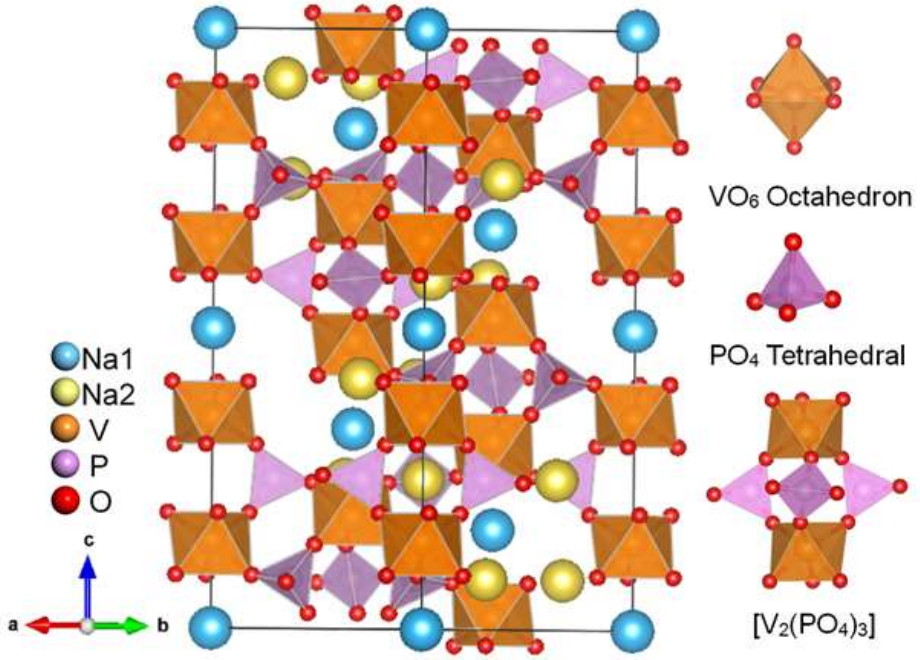

Figure S3. Structural model for $Na_3V_2(PO_4)_3$in our DFT calculations.

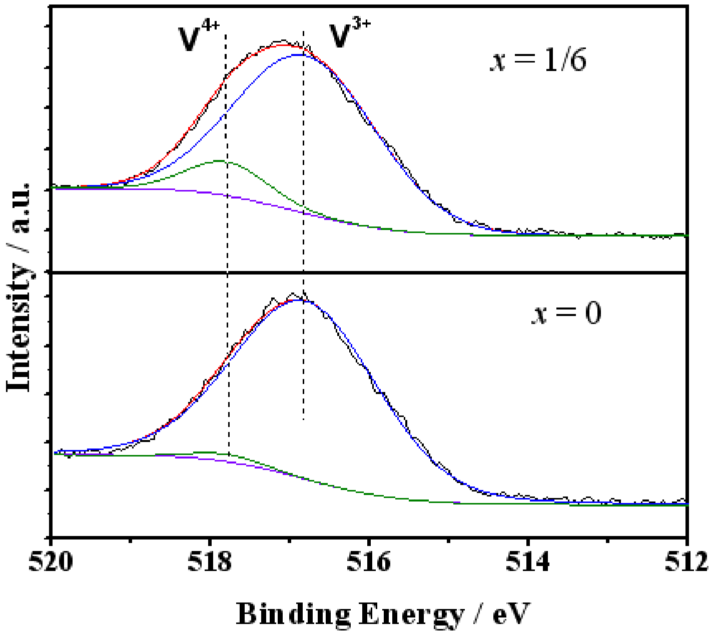

Figure S4. XPS of V 2p in $Na_3V_2P_{3-x}B_xO_{12}$ (x= 0 and x= 1/6)

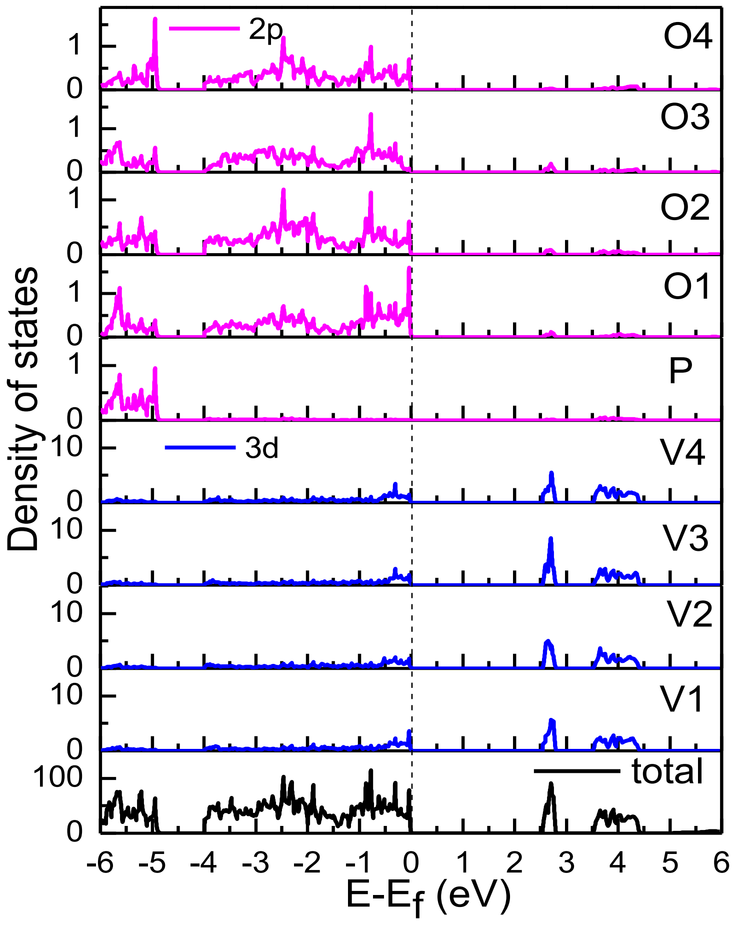

Figure S5. Calculated partial spin up density of states of $Na_3V_2(PO_4)_3$.

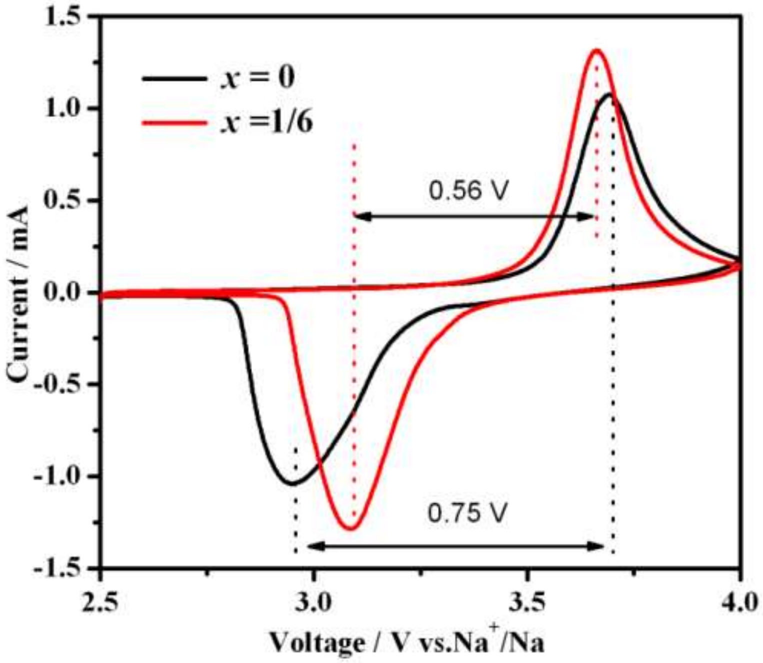

Figure S6. CV curves of the electrode at 0.5 mV s^{-1}.

Table S1 The structural parameters and band gaps (E_g)of pure $Na_3V_2(PO_4)_3$ and B doped $Na_3V_2(PO_4)_3$.

	a (Å)	*b* (Å)	*c* (Å)	*α*(°)	*β*(°)	*γ*(°)	V (Å^3)	E_g (eV)	
$Na_3V_2(PO_4)_3$	8.729	8.729	21.804	90	90	120	1438.73	—	Expt.
$Na_3V_2(PO_4)_3$	8.895	8.889	21.764	90	89.91	119.98	1490.52	2.565	Calc.
$Na_3V_2P_{3-1/6}B_{1/6}O_{12}$	8.862	8.848	21.749	90.1	89.75	120.02	1476.50	1.633	Calc.
$Na_3V_2P_{3-2/6}B_{2/6}O_{12}$	8.832	8.811	21.703	90.26	89.61	120.08	1461.43	1.622	Calc.
$Na_3V_{2-1/6}B_{1/6}(PO_4)_3$	8.856	8.849	22.293	90.33	89.45	120.02	1512.52	—	Calc.

Table S2. The bond distances (Å) of V-O in (a)$Na_3V_2(PO_4)_3$ and (b)$Na_3V_2P_{3-1/6}B_{1/6}O_{12}$

		i=1	i=2	i=3	i=4	i=5	i=6	Average
V1-Oi(Å)	(a)	2.007	2.123	2.060	2.009	2.054	2.068	2.054
	(b)	1.651	2.218	2.058	1.999	2.069	2.050	2.008
V2-Oi(Å)	(a)	2.072	2.035	2.113	2.071	2.019	2.029	2.057
	(b)	2.020	2.075	2.065	2.077	2.008	2.050	2.049
V3-Oi(Å)	(a)	2.009	2.068	2.007	2.060	2.123	2.054	2.054
	(b)	1.786	2.076	1.947	2.044	2.088	2.002	1.991
V4-Oi(Å)	(a)	2.113	2.019	2.029	2.035	2.071	2.072	2.057
	(b)	2.108	2.031	2.054	2.022	2.102	2.086	2.067
